# Partnering to proceed: scaling up adolescent sexual reproductive health programmes in Tanzania. Operational research into the factors that influenced local government uptake and implementation

**DOI:** 10.1186/1478-4505-8-12

**Published:** 2010-05-13

**Authors:** Jenny Renju, Maende Makokha, Charles Kato, Lemmy Medard, Bahati Andrew, Pieter Remes, John Changalucha, Angela Obasi

**Affiliations:** 1 National Institute for Medical Research, Mwanza Centre, P.O Box 1462, Mwanza, Tanzania; 2 Liverpool School of Tropical Medicine, Liverpool L3 5QA, UK; 3 African Medical and Research Foundation, PO Box 1482, Mwanza, Tanzania; 4 Social and Public Health Sciences Unit, Medical Research Council, Glasgow, UK

## Abstract

**Background:**

Little is known about how to implement promising small-scale projects to reduce reproductive ill health and HIV vulnerability in young people on a large scale. This evaluation documents and explains how a partnership between a non-governmental organization (NGO) and local government authorities (LGAs) influenced the LGA-led scale-up of an innovative NGO programme in the wider context of a new national multisectoral AIDS strategy.

**Methods:**

Four rounds of semi-structured interviews with 82 key informants, 8 group discussions with 49 district trainers and supervisors (DTS), 8 participatory workshops involving 52 DTS, and participant observations of 80% of LGA-led and 100% of NGO-led meetings were conducted, to ascertain views on project components, flow of communication and decision-making and amount of time DTS utilized undertaking project activities.

**Results:**

Despite a successful ten-fold scale-up of intervention activities in three years, full integration into LGA systems did not materialize. LGAs contributed significant human resources but limited finances; the NGO retained control over finances and decision-making and LGAs largely continued to view activities as NGO driven. Embedding of technical assistants (TAs) in the LGAs contributed to capacity building among district implementers, but may paradoxically have hindered project integration, because TAs were unable to effectively transition from an implementing to a facilitating role. Operation of NGO administration and financial mechanisms also hindered integration into district systems.

**Conclusions:**

Sustainable intervention scale-up requires operational, financial and psychological integration into local government mechanisms. This must include substantial time for district systems to try out implementation with only minimal NGO support and modest output targets. It must therefore go beyond the typical three- to four-year project cycles. Scale-up of NGO pilot projects of this nature also need NGOs to be flexible enough to adapt to local government planning cycles and ongoing evaluation is needed to ensure strategies employed to do so really do achieve full intervention integration.

## Background

**Figure 1:**
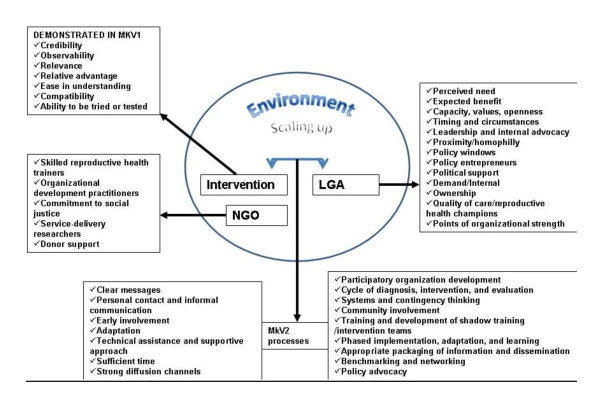
**A Model for scale up of interventions derived from diffusion of innovation and participatory organisational development theories**. Modified from Simmons et al 2002[6].

There have been various examples of successful small-scale interventions that aim to improve sexual and reproductive health and decrease HIV in young people in sub-Saharan Africa [1,2]. However, little is known about how best to implement such programmes on a scale great enough to significantly impact on adolescent sexual and reproductive health (ASRH) on this continent. Programme scale-up is complex: it is not simply a large-scale replication of a 'blue print' but the product of an interaction between an ideal intervention and the cultures, priorities and capacities of the structures through which it is scaled up [3-6]. Simmons et al. have developed models of scale-up and a series of recommendations based on an interrogation of public health case studies against diffusion of innovation and organizational development theories. These suggest that, in order to be sustainable, interventions must be scaled up through existing systems [3,6] and are most likely to succeed if intervention design is tailored to, and involves the structures through which scale-up is intended [6]. An integrated version of their model is presented in Figure 1.

However there is virtually no documentation of ASRH interventions that have been designed for scale-up through existing government systems in Sub-Saharan Africa. Evidence from other types of programmes (gender mainstreaming, health system reform and integration of STI programmes) in this setting describe challenges of low capacity, weak systems and a disconnect between national priorities and plans and district level activities [7-10]. Operational research helps to document and give a deeper understanding of the contextual factors that influence scaling up [5,11,12]. This type of evaluation plays an important role in the development and support of health systems. This paper presents the results of a series of evaluations that were conducted by an operational research team, which document and explain the scale-up of an ASRH intervention through a partnership between a non-governmental organization (NGO) and local government authorities (LGAs) in Mwanza Region, Tanzania. We examine the factors affecting intervention integration into LGA activities, we discuss how these relate to recommendations for sustainability highlighted above [6] (Figure 1) and make recommendations as to ways in which these findings can support local and national health systems.

### The intervention

MEMA kwa Vijana (MkV)(*Good things for young people*) is a multi-component ASRH intervention, developed and evaluated in 4 districts in Mwanza, Tanzania. It has been described in detail previously and remains one of the few interventions in sub-Saharan Africa to have been evaluated through the rigour of a community randomised trial (MkV1) [13-16]. In brief, from 1998-2001, teacher-led peer-assisted ASRH education, youth friendly services and community activities were implemented after in-depth training and with continued supervision from an NGO with over 50 years experience of developing and implementing public health interventions across Africa. The NGO also had a longstanding working relationship (over 10 years) with each of the four project districts. The intervention was designed in partnership with local and national government agencies specifically to be scaled up through government systems and was implemented with the support of the districts. Although there was no impact on biomedical outcomes, the intervention (implemented in 62 schools and 18 health facilities) showed substantial and sustained improvement in knowledge, reported attitudes and some reported sexual behaviours in the medium term (3 years) [16], and retained improved knowledge in the long term (8 years) [17].

Because of these benefits and in line with the first National Multisectoral Framework (NMSF1), which formed the overarching strategy for the HIV response at local government level, a ten-fold scale-up of the MkV intervention was commenced in 2004 to include all schools and health units in all 4 districts. In addition to district-wide implementation of the intervention, it was stipulated that the scale-up programme (MkV2) should specifically provide technical assistance to the LGAs in their multisectoral AIDS response and build LGA capacity through replication of the intervention.

**Figure 2:**
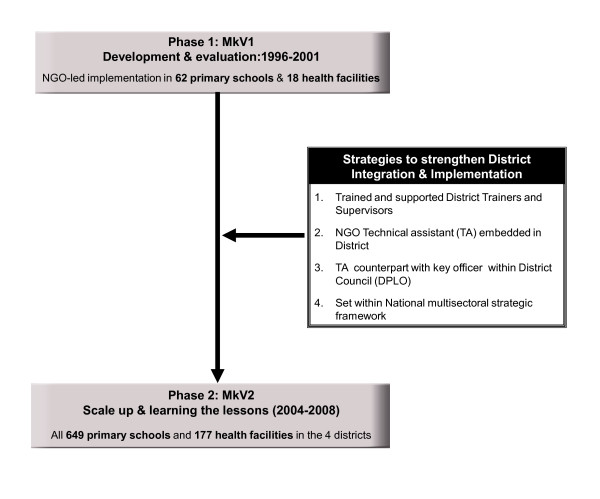
**Changes made to the MkV intervention in order to facilitate the transition from a NGO led project to an integrated, district led programme**.

**Figure 3:**
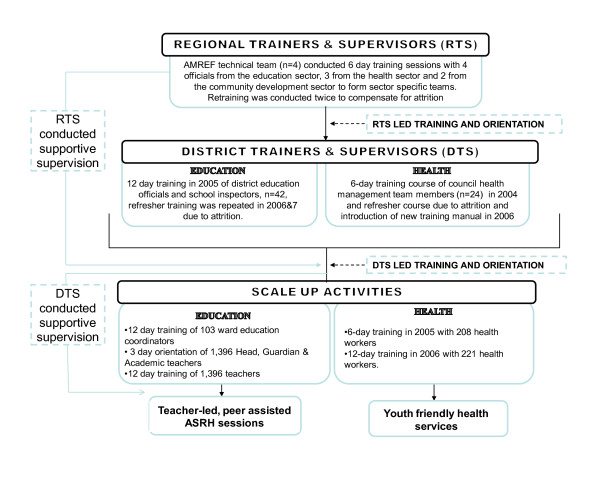
**Schematic diagram of the MkV2 cascade approach**.

To meet these goals MkV2 employed 4 overlapping strategies (Figure 2): (i) Officers from relevant LGA sectors were trained and supported as two teams (health and education) of district trainers and supervisors (DTS) to implement the intervention through a training cascade from regional to community level (Figure 3) and to supervise the teachers and health workers (HW) (The DTS were all district officials whose job descriptions included activities related to ASRH in respective sectors): (ii) District level project implementation was placed under the auspices of the District Planning Officer whose office is central to the running of the local government. Memoranda of understanding were signed to this effect and included phased cost sharing whereby the NGO would cover all costs of the training activities (year one) and the districts would incrementally support costs of supervision and routine maintenance (25% in year two, 50% in year three and 100% in year four): (iii) An NGO staff member was seconded as a technical assistant (TA) within each LGA. They were expected to work as a counterpart to the District Planning Officer and be situated within the planning office. Their specific role was to use the scale-up of MkV to support the LGA to develop a multisectoral AIDS plan, by building capacity across sectors, creating links between sectors and other organisations and networks in the area. (iv) Because of the congruence between the MkV intervention and the objectives and strategies of the NMSF1, MkV2 was explicitly identified as a vehicle to support the district implementation of NMSF1.

## Methods

### Study setting

Mwanza is the second most populous region in Tanzania and is situated along the southern shores of Lake Victoria. Thirty two percent of Mwanza's nearly 3 million population are between 10 and 24 years old [18]. The project was implemented in 4 of Mwanza's 8 districts. Within Tanzania the current government system is in the "dynamic process" of decentralisation [9,19]. Policies are made at the national level and the LGAs have two main functions: to ensure law and order and to plan for and implement activities aimed to stimulate the economic and social development of their districts. The districts submit annual budgets to the central government for approval, after a review process in parliament funds are then disbursed, often drastically modified.

#### Data collection

From 2005 to 2007, a team of researchers from the National Institute of Medical Research led by a research co-ordinator from Liverpool School of Tropical medicine, used multiple methods at regional, district and community level to explore the factors that influenced the uptake and implementation of the intervention via district systems. The research teams were independent of the NGO and District intervention and conducted a series of studies as follows:

### Key informant interviews with Regional and District Heads of Department

Four rounds of semi-structured key informant interviews (KII) were conducted with regional and LGA officials from June 2006 to August 2007 (Table 1). All the key informants were responsible for departments whose mandate, to varying degrees, involved issues pertaining to ASRH. Carefully developed topic guides were used to lead the discussions in three key areas: (i) the implementation of the NMSF(round 2); (ii) the integration of MkV2 into LGA systems (round 3), and; (iii) the effectiveness of the TA (round 4). The discussion topics are illustrated in Table 1.

**Table 1:** Coverage, respondents' details and discussion topics for the four rounds of data collection.

		Coverage		Respondent details		
			
Round	Date	Plan	Actual	Cadre	No	TOPICS
1	Aug-Sep '05	64	46 (72%)	District MkV implementers - Health	18	-training, planning, reporting and perceived successes and challenges of the first implementation year of MkV2
				District MkV implementers - Education	28	

2	Jun-Nov '06	44	41 (93%)	Regional officials	6	- Knowledge of key policies (source of information, content, structure)
				District officials	8	-Understanding of Multisectoral approaches
				District MkV implementers	23	-Activities in their department and relation to HIV/AIDS and ASRH
				NGO staff	4	-Expenditure profile in the district for AIDS/ASRH related activities. -Resource allocation (time) to MkV activities

3	May '07	116	69 (59%)	District officials	17	-Status of ASRH in districts -Understanding of MkV2 programme
				District MkV implementers	52	-Acceptance of MkV2 programme -resource allocation to ASRH in districts -structures/plans in place to continue implementation of MkV activities

4	Jul-Aug '07	46	44 (96%)	District officials	26	-planned, perceived and actual role of the MkV technical assistance (TA) model
				District MkV implementers	8	-Planned, perceived and actual benefits of the MkV TA model -Implementation status of the district AIDS response
				MkV team members	10	-Facilitating and inhibitory factors in the implementation of MkV and the TA model

#### Group discussions and workshops with the District trainers (DTS)

The DTS views and experience of intervention implementation were assessed in two rounds of 8 group discussions (one health and one education in each of the 4 districts) held in August-September 2005 and May 2007. Finally, between July and August 2007 eight interviews were conducted with a convenience sample of DTS to ascertain their views on the TA model.

#### Observations and participation in NGO or District-led MkV meetings

All members of the NGO team (including the seconded TA) met on a monthly basis to review progress and plans for future activities. TA and DTS co-ordinated annual evaluation and planning meetings at District level (n = 12). Between 2004 and 2008, independent researchers were present in approximately 80% of all district-based meetings and 100% of all Mwanza-based NGO-led monthly full team meetings with a dual role to observe (general interactions, issues arising in each district) and feedback preliminary analysis of research conducted to date. After each meeting researchers produced detailed reports following a pre-prepared guideline.

### Analysis

All interviews and group discussions were taped and transcribed. Transcripts and meeting reports were anonymised and coded in QSR NVIVO version 2.0 (Rouge Wave Software Inc., Yves Roumazeilles) and analysed using a thematic content approach. Factors influencing the implementation of the four strategies and intervention integration into district systems were identified and analysed. Data from each district were examined individually and then compared to ascertain differences and similarities within and between the districts.

These data were triangulated with results of the process evaluation of the district-led teacher and health worker training and facility level implementation. The latter (pre and post training questionnaires, informal interviews and training observations, teacher and HW interviews, mystery client studies and exercise book reviews) data is being presented in a series of separate papers and will only be mentioned briefly here.

### Ethics

This study was approved by the Tanzanian Medical Research Coordinating Committee. All interview participants gave written consent to participate and were free to withdraw at any time. Written informed consent was obtained from the patient for publication of this case report and accompanying images. A copy of the written consent is available for review by the Editor-in-Chief of this journal.

## Results

Eighty two of a target 118 KII, and 101 group discussions were conducted. Of the 36 KII that could not be performed, 20 officials were absent, 10 were too busy and six were on leave. However, owing to the repeated survey rounds, all heads of departments were included in the study at least once. After triangulation of the data from the multiple methods various thematic areas emerged: the implementation of trained and supported district teams; the integration of the programme into the LGA structure; the role of the technical assistants and finally the situation of activities within the National Multisectoral strategic framework. Outcomes within these thematic areas are presented here.

### 1. Implementation by trained and supported district teams

Facility level scale-up formed the backbone of MkV2. In four years the DTS successfully trained 1,396 teachers from 620 schools; 429 health workers from 179 heath facilities; and 103 ward education officers across four districts. The training led to improvements in knowledge, attitudes and reported self-efficacy among teachers (Scaling up a school-based sexual and reproductive health intervention in rural Tanzania; a process evaluation describing the implementation realities for the teachers, submitted) and in knowledge and attitudes among health workers (A process evaluation of the scale-up of a youth friendly health services initiative in Northern Tanzania, submitted).

Training observations, KII and FGD confirmed that DTS from all districts were active and committed to completing MkV2 activities and did not perceive the work (1-2 person-months/district/year) to be onerous.

"Aah I don't see that they have added additional work, because what is done as part of MEMA [MkV2] fits directly into the timetable of our everyday work. We don't have special time for MEMA activities, except maybe the training period which is normal as other training activities also take place."

District Trainer - Community Development

In one district involvement of two departments within the education sector (school inspection and education office) led to improved relations and co-ordination between the two offices. However the scale-up was hampered by high turnover of district staff, teachers and health workers. Further embedding the supervision rounds within the district structures meant that no follow up visits took place in some schools during the project period.

### 2. Integration into the LGA structure

The memorandum of understanding, signed by all districts, constituted a formal "political" or "legal" integration of the intervention. However, data on "psychological" (perceived ownership and congruence with LGA priorities), "operational" (day-to-day commitment of district time and personnel) and "financial" (actual use of LGA funds for MkV activities) integration were also examined. Such data were likely to indicate the quality of integration that is necessary for sustainable scale-up [6,20].

#### Psychological integration

The majority of DTS and heads of department listed ASRH as an implicit priority within other priority areas rather than a stand-alone concern. Respondents were most likely to list STDs, HIV or AIDS as the main problem (stated by 71% of head of department) with ASRH and its component issues (early pregnancies, young people's sexual behaviour, lack of youth friendly services for ASRH and the low education level amongst girls) being considered as related to these overall priorities.

Whilst districts generally welcomed the intervention, heads of department and DTS were much more likely to cite the different components of the intervention as opposed to its overall aims and objectives. In all districts MkV2 was viewed as an NGO-led project as opposed to a district-owned and led programme.

"It is strange that the entire district acknowledges that HIV/AIDS is a serious problem and yet they leave the programme entirely in the hands of the NGO"

Regional Trainer and Supervisor

"I cannot allow three teachers to leave from every school to take part in the 12 day training sessions any time after July. From July we are preparing for the standard seven exams and there is no questions concerning MkV in the exams, further the Ministry does not ask us if we are teaching MkV well. I will, however, be asked how many pupils passed or failed the standard 7 exams and why! ... this [after July] is a period to be closely following up how the teachers are teaching their sessions, it is not because we are reducing our collaboration with the project, it is only that we have to follow our [the district] work plan for our work."

District Education Officer's explanation to a Technical Assistant,

#### Operational integration

All LGA contributed DTS personnel to the programme ranging from 35 to 57 person-days per district per year. TA were granted office space, district staff were appointed to lead the activities within the districts and committed significant amounts of time. However, there were key differences between districts in the level of support and involvement of the leadership. For example in only one district did the district leaders regularly attend and chair MkV2 planning and evaluation meetings. TA participation in district political life is discussed below.

Turnover of key staff was a major factor affecting integration of the programme. In each district between 2004 and 2008 there was movement of district heads of department and DTS. Most notably the overall head of the district changed once in one district and three times in another. When such changes occurred there was always a substantial time lag between the old head of department leaving and the new one starting, which was covered by an interim appointee. The interim person was invariably hesitant to make any strategic decisions. These gaps also meant that there was no formal handover between office holders.

#### Financial integration

**Figure 4:**
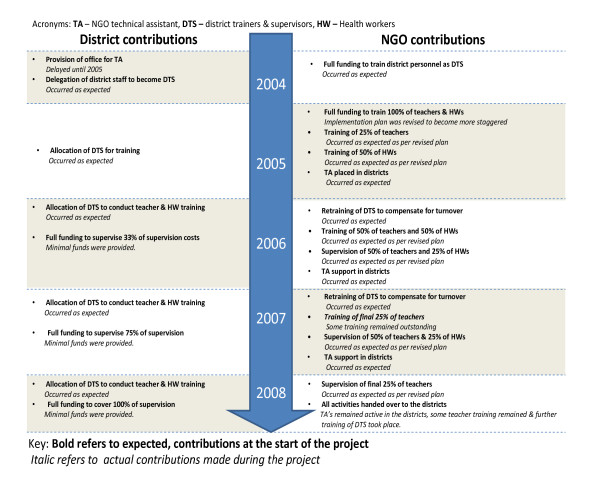
**Expected and actual contributions made by the District and the NGO over the five years of the project**.

Financial integration was severely hampered by three key factors: funding delays, mismatching of NGO and local government financial planning timetables and amendments made by the NGO to the overall project financial plan. Funds were disbursed annually from the donor to NGO headquarters in Dar es Salaam and thence to the NGO office in Mwanza, from where funds were distributed to the districts on a quarterly basis. However, each year from 2005 to 2007 there were delays in delivery of funds to the Mwanza office of between 2-7 months. This led to delays in disbursements to the district level. Secondly, recognition of the need for more gradual phase-in of training activities over the entire 4 years meant that the NGO was still committed to providing training funds in the final year of the scale-up. Although a more realistic model for implementation of scale-up activities at facility level, this departed from the initial proposal in which training was to have been completed in year 1 and 2 allowing district takeover of all project activities (i.e. supervision and maintenance costs) by project end (Figure 4). Finally, the annual financial planning in the LGA took place before that of the NGO. This, together with the funding delays discussed above, largely prevented formal inclusion of MkV2 activities into LGA annual plans prior to their submission for National level approval.

Notwithstanding this, two district governments allocated their own funds for MkV2 training of some health workers, and one of those also took the initiative to integrate ASRH activities within their existing Maternal and Child Health outreach services. The launch of the Ministry of Health youth friendly services manual and the involvement of the council health management team was influential in this process. A third district allocated funds for MkV2 training of teachers in new schools.

However no district made financial contributions to the level that was originally agreed during the inception of the project (Figure 4). The RTS questioned the lack of commitment:

"Council's commitment to program depends on who is there, e.g., dedication by District Executive Director. Activities may be delayed for trivial reasons and excuses including insufficient resources. But more could be done by the district."

Regional trainer, Community Development Sector

### 3. Technical Assistance

#### The technical assistant's role within the district

The TA's role *"to enhance capacity and facilitate broader support for the Districts to develop, co-ordinate and sustain a comprehensive multisectoral district AIDS response" *evolved during the life of the project. They possessed a range of skills and experience in working with LGAs, HIV, young people and in development work, and were oriented in MkV and the overall objective of their role (Table 2). However, initially, there was no specific job description or predefined outputs. The TAs were based in their respective districts and they built close links and good working relationships with the DTS. However, as the project evolved, in all but one district, TAs generally became linked to one sector (health or education). Links with the District Planning Officer were also weak, ostensibly because this, very senior official was very busy. But this may also have been because of attitudes to MkV2 (see below) or the lack of perceived need of planning office time if the TA was already present.

**Table 2:** Characteristics of the districts in reference to the technical assistants, implementers and outputs.

**District code**:	01	02	03	04
**Technical assistance**				
Number of TAs posted between 2004-2008	2	1	3	1
*Details of changes and TA background*				
TA1	Education background, district based, 2005-2007	Health background, district based 2005-2008	Health background, district based 2005-2006,	Education background, district based 2005-2008
TA2	Education background, district based 2007-2008	N/A	Education background, based in Mwanza, 2006-2007	N/A
TA3	N/A	N/A	Community development background, district based, 2007-2008	N/A

**Implementers**				
Number of DTS health originally trained	6	6	6	6
Turnover	0	1	2	0
Replacements trained	N/A	1	0	N/A
Number of DTS education originally trained	14	10	9	10
Turnover	4	1	1	3
Replacements trained	0	1	4	3

**Training outputs (proportion of target %)**				
Number of teachers	540 (77%)	477 (97%)	318 (100%)	390 (88%)
Number of health workers	125 (80%)	124 (77%)	90 (81%)	95 (89%)

Efforts were made to clarify TA roles by developing a terms of reference which reaffirmed the multi-sectoral nature of the position by strengthening links not only with the District Planning Office but also with the Council HIV/AIDS Coordinator. The latter was a new position created by the government in 2004 to coordinate the national multisectoral AIDS response. Finally, due to unavoidable staff transfers, during the project period, one district had three different TAs (two based in the district and one based in Mwanza but travelling to and from the district) and another district had two TAs (both district based) (Table 2).

Subsequent evaluation found that the TA effectively achieved some of the key tasks specified in the terms of reference. In particular they successfully: (i) facilitated the implementation of the MkV2 program (in terms of planning, training and supervision) and (ii) assisted in communication between LGA and the NGO. However their integration at district level, as measured by participation in different health fora, varied across the districts. For example, the TA was as a member of the council multisectoral AIDS committee in only one of the four districts.

Overall, many of the original limitations still remained at the end of the project. In 2007, TAs were still strongly linked to the NGO and assumed by districts to be responsible for the coordination of MkV2 activities. Further TAs found it difficult to let the districts "fail". Their monthly reports at the full team meetings demonstrated their belief that they were being judged on the coverage of the programme in the districts and felt that if an activity was not going well they had to take over rather than allow suboptimal implementation. Triangulation of the observations of the NGO monthly meetings and the interviews with the district heads of department indicated that only the first TA in one of the districts was reportedly able to stand back and allow their DTS to learn from their mistakes.

#### Perceptions of the TA model

When asked where the TA was most useful head of department provided a range of responses including during training (45%), planning (26%) and conducting supportive supervision (16%). Several (16%) heads of department (all from one district) reported that the presence of the TA ensured a degree of financial discipline concerning project funds.

The districts were most likely to view the TAs as implementers of the intervention as opposed to their intended role as facilitators and capacity builders. There were two notable exceptions: firstly in the district without a full-time TA, a greater number of respondents cited the TA as a facilitator and in another district where a number of respondents felt the TA's main role was to build capacity of the district officials.

When asked if the position of the TA was important for the programme more than 80% of all the respondents stated that without the TA the program could not have achieved high coverage. There were differences between the Districts and NGO as to whether the TA role should be maintained if the scale-up was to be repeated elsewhere. All key members of the NGO team and many of the TAs believed the role should continue, citing the situation in the district which did not have a district-based TA as justification.

"Where the TA was not permanently present, there was a big gap in that there was no link between the district and the NGO. Reports could not be easily accessed and information on program was lacking, as a result willingness on the part of council staff to implement MkV2 stalled."

MkV2 Project team member stated during a project meeting

"There is big difference between the district without a district-based TA and the other districts. In that district things did not move, implementation has been slow. There was no facilitation."

TA MkV2 stated during KII with the research team

However a third of heads of department from three districts (all that had district-based TAs) believed that a government official would be better placed to be trained and supported by the NGO to act as MkV TAs, arguing that that being an official government worker would increase the TAs level of involvement in district processes, and so increase the effectiveness of the model.

"TA is not involved because he is not on the District pay roll."

District head of department

"No one was born a TA; there is nothing so technical that others could not do...TAs are not God given, they were trained and assigned the tasks they perform, CHAC and others in the district could do the job."

District head of department

### 4. Situation of activities within the National Multisectoral Strategic Framework (NMSF)

As discussed, the NMSF was the core government strategy for the implementation of HIV related activities at local government level. However, KII highlighted key problems in the district level operation of the NMSF1 as follows: (i) whilst all the District respondents recognized the need for a comprehensive strategy for HIV and AIDS there was little to no understanding of the concept of a "multisectoral approach"; (ii) respondents felt that this strategy was imposed upon them from above with little or no consultation about what was happening on the ground and (iii) the methods used to disseminate and time allocated to implement the NMSF1 were inadequate.

Furthermore, the appointment of the Council HIV/AIDS Coordinator (CHAC) as NMSF1 coordinator within the district was problematic. In all four districts, this individual had previously been a desk/planning officer within the department for community development. The District AIDS Control Coordinator (DACC), on the other hand, was a senior health professional who had had overall responsibility for the prevention and treatment of HIV and AIDS within the health sector. In three of the four districts there was initial confusion over the roles of the CHAC and the DACC. This led to tensions at the individual and sector level, which were exacerbated by the lower rank of the CHAC and questions about the CHAC's technical ability to take on such a position, given their level of qualification and experience. In the fourth district it was clear that the two individuals understood their own roles clearly and the DACC was able to provide the CHAC with the technical support that he needed.

#### Differences between districts

The cascade training approach was successful in achieving a high coverage of schools and health facilities in all districts (75%-100%), though there were notable inter-district differences. Whilst the largest district (twice the size of district 3) reached the largest number of schools (n = 540) and health facilities (n = 125) its overall coverage was at the lower end of the range (77% and 80% respectively) (Table 2). There was little difference in coverage (in terms of number of schools/health facilities) between the districts which had a TA present throughout the project and those that had changes and/or periods without a TA. However districts which had a permanently based TA achieved a higher dosage (number of sessions taught) and more favourable training outcomes (teacher and health worker changes in knowledge, attitudes and self efficacy) (Scaling up a school-based sexual and reproductive health intervention in rural Tanzania; a process evaluation describing the implementation realities for the teachers, submitted and A process evaluation of the scale-up of a youth friendly health services initiative in Northern Tanzania, submitted).

## Discussion

This study adds to the limited documentation of factors that facilitate or inhibit the scaling up of interventions. The intervention had been designed with a view to scale-up though government systems and included most of the factors recommended for sustainable scale-up. A number of qualitative and quantitative methods were used to evaluate the integration of the scale-up of a model intervention through district systems. The range of methods strengthens the validity of the study. However, many of the techniques used within this study relied on reported data and are therefore subject to reporting bias. There was also no comparison group as all the original districts from the MKV1 trial were included in the scale-up. Conversely, the use of researchers who were independent of the implementation process decreased the likelihood of bias, as did the use of direct observations of trainings and meetings and the triangulation of multiple methods repeated over time.

Overall this study highlights important operational issues in the integration of large scale multi-component ASRH interventions into district systems and the effectiveness of the particular strategies used in this project. Firstly, the cascade model, supported by the TA, was successful in scaling up the intervention to 75-100% of the intended schools and health facilities in each of the four districts. The DTS were motivated and demonstrated ownership, skills and flexibility in their implementation activities. This underpinned the operational integration of the programme.

However, in order to be effective, integration of a project into an existing system must take place at several (formal, operational, financial and psychological) levels. Perceived need for the intervention within the system is key to the psychological integration of the programme [4,6,21]. The status of ASRH as a subsidiary rather than an overarching priority was a barrier to the integration as was the perpetuation of the traditional donor-NGO-District pathways of funding and administration. The latter served to maintain MkV2's status as an external project within the districts and undermined financial integration by limiting district ownership, ability and intention to make financial contributions to the implementation of these activities.

Simmons *et al*. [6] discuss the need for resources and user systems to be similar. MkV2 highlighted that this does not only refer to financial resources, control of which is a crucial determining factor in the ownership of a project, but also in terms of the proposed timeframe. The implementation speed of MkV2 (100% coverage in three years) far outstripped the normal pace of the scale up of other government led activities. It is likely that this haste presented another barrier to psychological integration, by distinguishing MkV2 from other district activities, further underlining its status as an project and increasing the dependency on the TAs.

Scale-up also needs to overcome capacity constraints [6,9,21-23]. Supportive technical assistance is seen as key and here the placement of the TA within the district worked to build capacity and the study suggests that where the TA was present the teacher and health worker training led to more significant changes in knowledge and attitudes. However, genuine capacity building takes time that is likely to exceed the typical three to four year donor funded project cycle. Our study suggest that the short-term benefits of the embedded TA model used in this project, may need to be offset against the longer term costs of delayed or impaired integration. Although, in the one district where the TA was more able to 'stand back' the DTS were able and willing to implement the scale-up activities to a high standard and the LGA set aside funds (although not to the level originally agreed) for scale-up activities.

Overall therefore, although the TA model was explicitly intended to build capacity for integration into district activities, it may have been a barrier to it. An alternative to the secondment of an NGO employee would have been the training of an LGA employee to occupy the role of the TA. However this would have required substantial additional time and resources in terms of selection and training. These were not available within the project time frame.

The experience presented here has demonstrated that capacity was not only important in the system to which responsibility was transferred [6,23] (i.e. to the districts) but also the system from whence it originated (i.e. from the NGO). The NGO and in particular the TAs responsible in this arrangement were much more comfortable with the management and implementation of projects than their intended advisory and facilitative roles. The findings support the notion that capacity building is more than just the training at the level of the individual [23]. In reality DTS training was efficient and effective. DTS turnover did not significantly affect facility level implementation and training activities were not perceived as onerous. However, in contrast to implementation, *sustainability *really depends on structural aspects of systems. In the case of MkV2 this would have meant strategies to address an ingrained "project" mentality, (i.e. a tendency to see key activities as being the responsibility of NGO projects) and donor dependency on the part of both the NGO and LGA. Further it would mean that there would need to be much more flexibility on the part of the NGO to be able to adapt to districts planning and reporting cycles.

Effective leadership and motivated individuals are essential factors in the transfer of ownership and implementation from one entity to another [6,10,21]. The regular change of leadership and key personnel both within the government and NGO was a challenge, threatening the continuity of activities and level of understanding of the project's aims and activities. Any programme of this nature needs strategies to overcome this turnover.

Policy windows should ideally enhance scale-up by underpinning psychological and operational integration [6]. The content and timing of NMSF1 made it an apparently ideal policy within which to nest the MkV2 scale-up. However, limitations in its conception and dissemination of NMSF1 undermined its utility at LGA level. This included a lack of understanding of clear district multi-sectoral AIDS plans, uncertainty about personnel roles and lack of ownership at district level. The fact that MkV2 was seen by the LGA as a major part of the districts' multi-sectoral AIDS response as opposed to a catalyst for the formulation of this response was symptomatic of a disconnect between policy formulation and implementation [7,10,24].

A bottom-up approach to development is important to ensure interventions are context specific [21]. The MkV intervention was developed through local partnerships in line with national guidelines and with input from National bodies and initiatives [14]. This research supports the observation that when a system is not completely decentralized, as is the case in Tanzania, [9] programmes need to work both from the bottom up and the top down in order to build the capacity within the system and bridge the disconnect between policy formulation and its implementation [25].

## Conclusions

Large-scale public health problems require large-scale responses: proven interventions need to be scaled up in order to increase coverage. This project successfully achieved a 10 fold increase in intervention scale over four years whilst maintaining good standards of implementation through district systems. However, whilst NGO assistance at local government level was successful in supporting operational scale-up, our research suggested that psychological and financial integration were hampered by high rates of senior staff turnover, persistent strategic and financial control by the NGO and limited understanding and acceptance of the overarching policy framework at LGA level. In addition to demonstrating that full financial integration of scale-up activities into LGA plans was not achieved, we provided possible explanations as to why. Future scale-ups of this nature must strengthen and sustain the districts' active involvement in the operational management of scale-up. Programming cycles should therefore be lengthened and accept more modest initial outputs in order to ensure development, not only of implementation capability among district based staff, but of genuine financial and psychological integration into district systems.

## Competing interests

The authors declare that they have no competing interests.

## Authors' contributions

All authors have read and approved the final manuscript:

JR was the research coordinator for the scale-up of MEMA kwa Vijana (MkV2), she conceptualised the study and supervised the data collection and performed the final analysis and write-up of all the components of this study and manuscript.

MM worked with the lead author to conceptualize the study, and drafted sections of various iterations of this manuscript and the abstract of the final version.

CK led the implementation of the health component within AMREF; he supported the design and implementation of the research and commented on various drafts of this paper.

LM and BA were responsible for the data collection of all components of this study; they were involved in the design and co-drafted the first iteration of this manuscript.

PR provided ongoing input into the data collection and interpretation as well as significant input into the preparation of the final manuscript by critically reviewing previous drafts.

JC was co-Principal Investigator of the MkV2 and supported the research on all phases of MkV2.

AO was a co-Principal Investigator of the MkV2, oversaw the design, implementation and interpretation of all the studies in this paper, and contributed substantially to the various drafts of the manuscript.

## Abbreviations

**AMREF**: African Medical and Research Foundation; **ASRH**: adolescent sexual and reproductive health; **CHAC**: Council HIV/AIDS coordinator; **DACC**: district AIDS control coordinator; **DPLO**: district planning officer; **DTS**: District trainers and supervisors; **GD**: group discussion; **HW**: Health worker; **KII**: key informant interview; **LGA**: local government authorities; **NGO**: Non-governmental organisation; **NMSF1**: the first National Multisectoral Strategic Framework (2004-2007); **MkV2**: MEMA kwa Vijana (phase 2); **RH**: reproductive health; **TA**: technical assistant.
